# Hybrid Arch for Acute Type A Aortic Dissection: When to Deploy the Endograft? Debate: Frozen versus Staged?

**DOI:** 10.1055/s-0038-1669415

**Published:** 2018-11-19

**Authors:** Jehangir J. Appoo, Akash Fichadiya, Holly N. Smith, Vamshi K. Kotha, Eric J. Herget, Alexander J. Gregory, Wilson Y. Szeto

**Affiliations:** 1 Division of Cardiac Surgery, Libin Cardiovascular Institute, University of Calgary, Cumming School of Medicine, Calgary, Canada; 2 Department of Diagnostic Imaging, University of Calgary, Cumming School of Medicine, Calgary, Canada; 3 Department of Anaesthesia, University of Calgary, Cumming School of Medicine, Calgary, Canada; 4 Division of Cardiac Surgery, University of Pennsylvania School of Medicine, Philadelphia, Pennsylvania

**Keywords:** aortic dissection, TEVAR, hybrid arch

## Abstract

Advances in open and endovascular techniques have resulted in novel approaches to repair of acute Type A aortic dissection. Hybrid arch procedures involve open arch resection and stent grafting of the descending aorta with stent graft insertion in one of two ways: Frozen or Staged. In this article, pros and cons of the two different paradigms of emerging hybrid arch techniques for acute Type A aortic dissections are discussed.

## Introduction

Current debate regarding surgical repair of acute Type A aortic dissection (ATAAD) centers around whether an aggressive approach to the aortic arch should be undertaken, compared with an open distal anastomosis known as a hemi-arch. There are no randomized trials comparing these two approaches. While the results of surgery for ATAAD have improved over time, the most recent large registry datasets published in 2015 suggest a persistently high operative mortality of 15 to 20%. 1 2 Furthermore, patients who survive the initial repair of ATAAD have decreased long-term survival, with 10-year survival rates reported as low as 46%. 3 Recent advances in open and endovascular surgical techniques have led to novel interventions aiming to improve both short- and long-term outcomes in this challenging patient population. Hybrid arch procedures involve open arch resection and stent grafting of the descending aorta. The stent graft may be inserted in one of two ways: early—during circulatory arrest or staged—after separation from cardiopulmonary bypass (CPB) with the use of fluoroscopy. In this debate, we discuss pros and cons of different emerging hybrid arch techniques for ATAAD.

## Hybrid Arch for ATAAD

A variety of surgical techniques have been proposed under the broad classification of “hybrid arch” repair, which involves surgical total or partial arch replacement and endovascular aortic stent graft deployment to treat a more extensive portion of the dissected aorta. These techniques raise the question of whether a more complex surgery can produce better outcomes.

The goals of hybrid arch surgery in ATAAD are to resect the primary intimal tear and seal tears extending beyond the transverse aortic arch, as well as to cause false lumen obliteration of the descending thoracic aorta. Theoretical benefits include reductions in early malperfusion, in late distal aortic dilatation, in need for late aortic reintervention, and in mortality. These hybrid techniques may have particular value in patients with end-organ malperfusion, as studies have shown that this population has a fivefold increase in operative mortality compared with ATAAD without organ malperfusion. 4 Recent guideline position statements are recognizing the utility of a more extensive surgical approach to treat ATAAD. The 2014 European Society of Cardiology Guidelines on Diagnosis and Treatment of Aortic Diseases recommends that in patients with ATAAD and organ malperfusion, a hybrid approach should be considered (Class IIa, level of evidence B). 5 The 2016 Canadian Joint Position Statement on Open and Endovascular Thoracic Aortic Surgery recommends an extended arch technique be considered for ATAAD under the following circumstances: primary intimal tear in the arch or beyond, significant aneurysmal disease of the arch, distal malperfusion, concomitant descending aortic aneurysm, young patients, and patients with connective tissue disease. 6 While traditionally surgeons have been hesitant to increase the extent of arch surgery in ATAAD, the existing literature suggests that hybrid arch operations, when performed by experienced aortic surgeons, may be accomplished without an increased risk of morbidity and mortality. A recent systematic review of > 2,100 patients from 38 publications revealed a surprisingly low pooled operative mortality of 8.6% across extended arch techniques for ATAAD. 7

If hybrid arch procedures are going to be part of the future armamentarium of surgery for ATAAD, the optimal technique of stent graft deployment needs to be determined. During these hybrid arch procedures, after surgical arch replacement with a Dacron graft, the stent graft can be inserted in one of two ways: (1) through the open arch during circulatory arrest or (2) staged after separation from CPB using fluoroscopy and standard thoracic endovascular aortic repair (TEVAR) techniques.

Industry has brought several new tools to market to facilitate hybrid arch procedures. Combination Dacron and stented grafts such as the Evita (JOTEC GmbH) and Thoraflex (Vascutek Ltd) prosthesis facilitate the insertion of a stent graft during circulatory arrest. Branched arch prostheses like the Bavaria and Lupiae grafts (Vascutek Ltd) facilitate creation of a proximal landing zone for the insertion of a stent graft post CPB. While both techniques aim to accomplish both exclusion of arch tears, as well as expansion of the distal true lumen and obliteration of the proximal thoracic false lumen, each has its own advantages and limitations ( Table 1 ).

**Table 1:** Advantages of early frozen elephant trunk versus late stent graft approaches

**One-stage FET advantages:**
No endo skills required
No fluoro or hybrid room required
No nephrotoxic agents (dye)
Cuff facilitates distal anastomosis
Clinical experience already accumulated
**Two-stage stent graft advantages:**
Radiographic confirmation of proximal and distal landing zones
Endoleak detection
Confirmation of no new tears created
Assessment of vessel patency and false lumen obliteration
Purpose designed grafts (including potential tapered or for multiple “tromboned” grafts)
Greater descending coverage possible
Resolution of visceral, renal, and peripheral malperfusion can be confirmed
Additional stent grafts or bare metal stents may be deployed, if necessary
No increase in DHCA time
BP can be kept higher (no anastomoses to protect) thus improving spinal cord perfusion
Facilitates early extubation and neurologic assessment

Abbreviations: BP, blood pressure; DHCA, deep hypothermic circulatory arrest; FET, frozen elephant trunk.

## “Frozen” Elephant Trunk

**Fig. 1:**
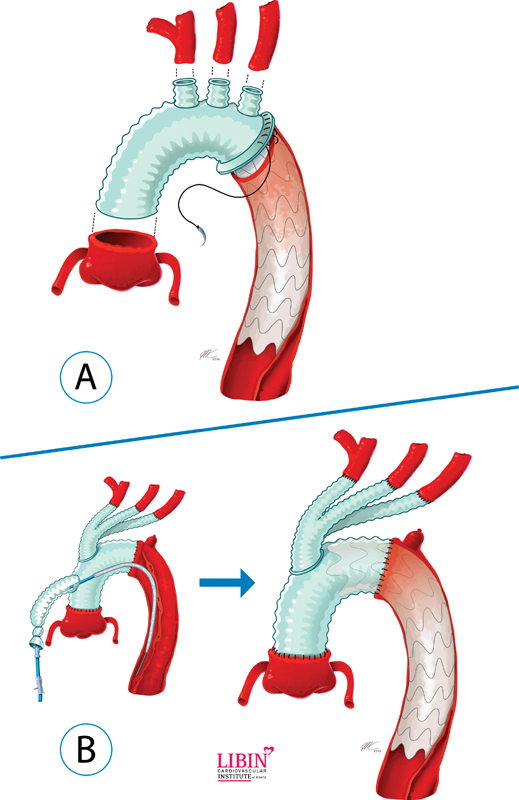
( **A** ) Frozen stented elephant trunk technique: a single-piece combined stent graft and branched Dacron graft is used. Stent graft is deployed antegrade through the open arch during hypothermic circulatory arrest. Surgical anastomoses are constructed (1) in the distal arch, (2) by end-to-end anastomosis to the three-arch vessels, and (3) and at the level of the sinotubular junction. ( **B** ) Zone 2 hybrid arch with staged endograft insertion: distal arch anastomosis is constructed at the level of the left subclavian artery; the ostium of the three-arch branches is transposed to a more proximal location leaving a 2 to 4 cm length of Dacron as a robust landing zone for the endograft. The stent graft is deployed either antegrade via a perfusion limb of the implanted Dacron graft or retrograde from the femoral artery after separating from cardiopulmonary bypass with the use of intraoperative fluoroscopy. (Reproduced with permission from www.aorta.ca .)

The “frozen stented elephant trunk” technique has been widely published in the literature and has its own accepted acronym “FET.” The basis of this technique is antegrade deployment of the descending aortic stent graft through the open arch during hypothermic circulatory arrest ( Fig. 1A ). This can be done over a wire inserted from the groin or simply advanced into the true lumen without a wire. Some surgeons prefer to visually inspect distal tears with a bronchoscope inserted through the arch, whereas others deploy “blindly” into what appears to be the true lumen.

Advantages of this technique over deployment post CPB include simplicity, in that a dedicated endovascular skill set is not required. In addition, this technique does not require the use of portable fluoroscopy equipment or a hybrid operating suite. As no pre- or postdeployment angiogram is done, potentially nephrotoxic intravenous contrast is not required. The large sewing collar that comes with these hybrid prostheses is advantageous, as it may facilitate an easier distal anastomosis. Finally, there is more data on this technique, as several large case series have shown “acceptable” morbidity and mortality, with potential long-term survival benefit. 8

## Staged Endograft Insertion Post CPB

This technique involves endovascular stent graft deployment in a traditional TEVAR fashion with the use of fluoroscopy to identify landing zones. 9 The arch is first replaced with a branched arch graft in a fashion that creates a robust proximal landing zone to set up future TEVAR. The endograft can be inserted after separation from CPB ( Fig. 1B ) at time of the index operation or days later. 10 Stent graft deployment may be performed either antegrade from the open chest via a perfusion limb of the implanted Dacron graft or in a retrograde fashion via the femoral artery. After separation from CPB, assessment can be made as to whether the arch replacement is adequate by itself to resolve distal malperfusion or whether a stent graft is required to expand the true lumen. If fluoroscopy confirms resolution of malperfusion, further intervention can be done in a delayed fashion.

**Fig. 2:**
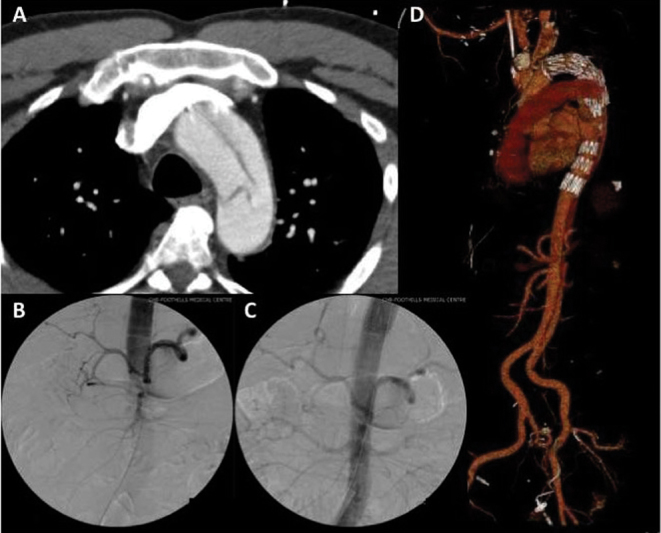
Case example of hybrid arch with warm stent draft technique in acute Type A aortic dissection (ATAAD) with malperfusion. ( **A** ) Axial computed tomography (CT) image of a 49-year-old male presenting with ATAAD. Note the large primary intimal tear distal to the left subclavian artery. ( **B** ) Intraoperative angiogram done via a pigtail in true lumen of descending aorta after completion of surgical zone 2 arch repair and prior to deployment of endograft. Note the lack of contrast filling the distal aorta, visceral, and renal vessels. ( **C** ) Intraoperative angiogram after deployment of endograft in retrograde fashion demonstrates impressive restoration of flow and radiologic confirmation of resolution of malperfusion. ( **D** ) Postoperative volume-rendered CT image demonstrating obliteration of false lumen in proximal descending aorta. The false lumen persists distal to the stent graft. The distal aorta is well perfused.

Advantages of deployment post CPB include radiographic confirmation of adequate proximal and distal sealing zones, detection of endoleaks, confirmation of no new tears (stent-induced new entry tear), 11 and assessment of false lumen obliteration and branch vessel patency. Use of traditional TEVAR skills allows insertion of tapered devices or devices of different sizes implanted in a tromboned fashion to obtain adequate seal proximally, without oversizing the distal landing zone in dissected aorta. Extent of descending aortic coverage can be individualized to the clinical setting. Importantly, in the setting of acute dissection, resolution of visceral, renal, or peripheral malperfusion can be confirmed on the operating room table with the use of fluoroscopy. The surgeon may then deploy additional stent grafts, or bare metal stents, if deemed necessary ( Fig. 2 ). As the stent graft is placed following separation from CPB, there is no increase in hypothermic circulatory arrest time necessary to deploy through the open arch. While both strategies carry a risk of spinal cord ischemia, a staged approach may protect against the risk of spinal cord ischemia as blood pressure can be kept higher and the patient extubated early to facilitate neurologic assessment. Finally, stent grafts are generally made of nitinol, which is a temperature-sensitive material that does not deploy to the determined diameter unless at normothermia.

## Conclusion

As the surgical community recognizes ATAAD as a diffuse process that can affect every organ system, there is a move toward expanding past the most acutely life-threatening complication of proximal aortic repair and treating more segments of the aorta, and addressing clinically significant branch vessel involvement. In the discussion above, we have compared the advantages of graft deployment during circulatory arrest with stent graft deployment post CPB. At our centers, the ability to assess our repair with intraoperative fluoroscopy has led to therapeutic changes in the management of our patients with ATAAD. Our personal bias is to use intraoperative fluoroscopy at the time of hybrid arch repair for ATAAD and not to deploy the endograft blindly. Others have also advocated use of a hybrid room for ATAAD, whereby all diagnostic and therapeutic measures are available. There are concerns about endovascular stent grafts being used by clinicians who do not have the tool kit to diagnose and manage adverse outcomes at the distal landing zone. However, we recognize that the technique of FET can be more widely adapted by cardiac surgeons doing operations on an emergency basis while on call. Extended arch operations can be very challenging for the general cardiac surgeon who is usually performing these uncommon operations at inopportune times

Our opinion is that results of endovascular stent graft use in ATAAD may be optimal if performed by aortic surgeons who can identify the pathology, know the treatment options, and are capable of both open and closed techniques.

## Future Areas of Research

Hybrid arch repair techniques have the potential to improve both perioperative and long-term outcomes in a challenging patient population. The goal of operative intervention for ATAAD is to minimize short-term mortality, while hopefully providing a close to normal life expectancy and quality of life in the long term. However, it remains to be seen which techniques will ultimately produce the best results for individual patients. A classification system of hybrid arch techniques will be required to compare outcomes. We propose that this classification system pays attention to method of stent graft deployment: during circulatory arrest or staged post-CPB. Future trials of surgery for ATAAD may address “hemi-arch” versus “total arch & descending stent graft” or “frozen elephant trunk” versus “staged endograft.”

## References

[1] Pape L A Awais M Woznicki E M Presentation, diagnosis, and outcomes of acute aortic dissection: 17-year trends from the international registry of acute aortic dissection J Am Coll Cardiol 2015 66 04 350 358 26205591 10.1016/j.jacc.2015.05.029

[2] Czerny M Schoenhoff F Etz C The impact of pre-operative malperfusion on outcome in acute type A aortic dissection: results from the GERAADA registry J Am Coll Cardiol 2015 65 24 2628 2635 26088302 10.1016/j.jacc.2015.04.030

[3] Geirsson A Bavaria J E Swarr D Fate of the residual distal and proximal aorta after acute type A dissection repair using a contemporary surgical reconstruction algorithm Ann Thorac Surg 2007 84 06 1955 1964, discussion 1955–1964 18036916 10.1016/j.athoracsur.2007.07.017

[4] Geirsson A Szeto W Y Pochettino A Significance of malperfusion syndromes prior to contemporary surgical repair for acute type A dissection: outcomes and need for additional revascularizations Eur J Cardiothorac Surg 2007 32 02 255 262 17500002 10.1016/j.ejcts.2007.04.012

[5] Erbel R Aboyans V Boileau C 2014 ESC Guidelines on the diagnosis and treatment of aortic diseases: document covering acute and chronic aortic diseases of the thoracic and abdominal aorta of the adult Eur Heart J 2014 35 41 2873 2926 25173340 10.1093/eurheartj/ehu281

[6] Appoo J J Bozinovski J Chu M W Canadian Cardiovascular Society/Canadian Society of Cardiac Surgeons/Canadian Society for Vascular Surgery Joint Position Statement on Open and Endovascular Surgery for Thoracic Aortic Disease Can J Cardiol 2016 32 06 703 713 27233892 10.1016/j.cjca.2015.12.037

[7] Smith H N Boodhwani M Ouzounian M Classification and outcomes of extended arch repair for acute type A aortic dissection: a systematic review and meta-analysis Interact Cardiovasc Thorac Surg 2017 24 03 450 459 28040765 10.1093/icvts/ivw355

[8] Pochettino A Brinkman W T Moeller P Antegrade thoracic stent grafting during repair of acute DeBakey I dissection prevents development of thoracoabdominal aortic aneurysms Ann Thorac Surg 2009 88 02 482 489, discussion 489–490 19632398 10.1016/j.athoracsur.2009.04.046

[9] Appoo J J Gregory A J Fichadiya A Kotha V K Herget E J Zone 2 arch replacement and staged thoracic endovascular aortic repair for acute type A aortic dissection Ann Thorac Surg 2017 104 03 e299 e301 28838535 10.1016/j.athoracsur.2017.05.039

[10] Marullo A G Bichi S Pennetta R A Hybrid aortic arch debranching with staged endovascular completion in DeBakey type I aortic dissection Ann Thorac Surg 2010 90 06 1847 1853 21095323 10.1016/j.athoracsur.2010.07.077

[11] Weng S H Weng C F Chen W Y Reintervention for distal stent graft-induced new entry after endovascular repair with a stainless steel-based device in aortic dissection J Vasc Surg 2013 57 01 64 71 23141675 10.1016/j.jvs.2012.07.006

